# Vesicular Acetylcholine Transporter Alters Cholinergic Tone and Synaptic Plasticity in DYT1 Dystonia

**DOI:** 10.1002/mds.28698

**Published:** 2021-06-26

**Authors:** Annalisa Tassone, Giuseppina Martella, Maria Meringolo, Valentina Vanni, Giuseppe Sciamanna, Giulia Ponterio, Paola Imbriani, Paola Bonsi, Antonio Pisani

**Affiliations:** ^1^ Laboratory of Neurophysiology and Plasticity IRCCS Fondazione Santa Lucia Rome Italy; ^2^ Department of Brain and Behavioral Sciences University of Pavia Pavia Italy; ^3^ IRCCS Mondino Foundation Pavia Italy

**Keywords:** acetylcholine, striatum, cholinergic interneurons, vesicular acetylcholine transporter, acetylcholinesterase

## Abstract

**Background:**

Acetylcholine‐mediated transmission plays a central role in the impairment of corticostriatal synaptic activity and plasticity in multiple DYT1 mouse models. However, the nature of such alteration remains unclear.

**Objective:**

The aim of the present work was to characterize the mechanistic basis of cholinergic dysfunction in DYT1 dystonia to identify potential targets for pharmacological intervention.

**Methods:**

We utilized electrophysiology recordings, immunohistochemistry, enzymatic activity assays, and Western blotting techniques to analyze in detail the cholinergic machinery in the dorsal striatum of the Tor1a^+/−^ mouse model of DYT1 dystonia.

**Results:**

We found a significant increase in the vesicular acetylcholine transporter (VAChT) protein level, the protein responsible for loading acetylcholine (ACh) from the cytosol into synaptic vesicles, which indicates an altered cholinergic tone. Accordingly, in Tor1a^+/−^ mice we measured a robust elevation in basal ACh content coupled to a compensatory enhancement of acetylcholinesterase (AChE) enzymatic activity. Moreover, pharmacological activation of dopamine D2 receptors, which is expected to reduce ACh levels, caused an abnormal elevation in its content, as compared to controls. Patch‐clamp recordings revealed a reduced effect of AChE inhibitors on cholinergic interneuron excitability, whereas muscarinic autoreceptor function was preserved. Finally, we tested the hypothesis that blockade of VAChT could restore corticostriatal long‐term synaptic plasticity deficits. Vesamicol, a selective VAChT inhibitor, rescued a normal expression of synaptic plasticity.

**Conclusions:**

Overall, our findings indicate that VAChT is a key player in the alterations of striatal plasticity and a novel target to normalize cholinergic dysfunction observed in DYT1 dystonia. © 2021 The Authors. *Movement Disorders* published by Wiley Periodicals LLC on behalf of International Parkinson and Movement Disorder Society

## Graphical abstract

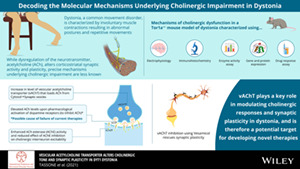

December Infographic 1 : Vesicular acetylcholine transporter alters cholinergic tone and synaptic plasticity in DYT1 dystonia

Dystonia is a common movement disorder characterized by sustained, repetitive muscle contractions, twisting movements, and abnormal postures.^1^ Deletion of three base pairs (ΔGAG) in the *TOR1A* gene results in the loss of a glutamic acid in the C‐terminal of the protein torsinA that is associated to early‐onset generalized dystonia (DYT1).^2^ Currently, the precise function of torsinA remains unclear, although it plays a role in multiple cellular activities, including protein folding, protein quality control of the endoplasmic reticulum, control of secretion, lipid metabolism, trafficking, degradation, and nuclear envelope dynamics.^3^, ^4^, ^5^, ^6^, ^7^

Anticholinergic drugs are among the few pharmacological options for medical treatment of DYT1 dystonia,^8^, ^9^ although their use is impeded by a number of serious adverse effects. The rationale behind the use of anticholinergic agents relies on the imbalance between striatal dopamine and acetylcholine (ACh), which results in an increased cholinergic tone.^10^, ^11^, ^12^ Although cholinergic interneurons (ChIs) represent a minority of striatal neuronal population, they produce an extensive and dense innervation, providing the highest regional level of ACh and cholinergic markers in the brain and playing a central role in the integration of corticostriatal and thalamostriatal inputs with dopaminergic nigrostriatal innervation.^13^, ^14^ In normal conditions, striatal cholinergic tone is downregulated either by acetylcholinesterases (AChE), ACh‐degrading enzymes, or by the activation of both muscarinic M2/M4 autoreceptors (M2/M4 mAChR) and dopamine 2 receptors (D2R). Indeed, D2R activation normally reduces the frequency of spontaneous firing of ChIs and inhibits the release of ACh.^15^, ^16^, ^17^ There is now robust evidence for a fundamental alteration of D2R signaling in the DYT1 mouse striatum consisting in an excitation, rather than inhibition, of the firing frequency of striatal ChIs.^18^, ^19^ The loss of the mutual control between striatal dopamine and ACh and the subsequent neurochemical imbalance are responsible for the impairment of bidirectional corticostriatal synaptic plasticity. Indeed, multiple DYT1 dystonia rodent models showed significant abnormalities in corticostriatal long‐term synaptic plasticity, exhibiting the loss of both long‐term depression (LTD) and synaptic depotentiation,^20^, ^21^, ^22^, ^23^, ^24^ as well as an enhanced long‐term potentiation.^20^, ^21^ Accordingly, these synaptic plasticity deficits could be compensated either by lowering ACh content or by antagonizing M1 muscarinic receptors.^20^, ^21^, ^23^, ^25^

Despite such extensive evidence, the nature of ACh dysregulation remains to be clarified. Here, we utilized a multidisciplinary approach to measure the possible changes in cholinergic markers, and their functional impact in the pathophysiology of dystonia, in the Tor1a null (Tor1a^+/−^) mouse model.^26^ These mice show a decrease in torsinA levels,^19^ as well as electrophysiological and molecular alterations common to multiple rodent models of DYT1 dystonia (for a review, see 27, 28, 29). In Tor1a^+/−^ mice, we identified a significant alteration in the expression of the striatal vesicular acetylcholine transporter (VAChT), which loads ACh into the presynaptic vesicles before its release through exocytosis. Blockade of VAChT restored long‐term synaptic depression (LTD) alterations, indicating its functional relevance. Altogether, our results indicate that VAChT is a potential target for pharmacological intervention.

## Materials and Methods

### Mouse Model

Male Tor1a^+/−^ null mice^26^ (9‐ to 12‐week‐old, B6; 129‐Tor1atm1Wtd/J, 006251 The Jackson Laboratory) and their wild‐type littermates were used for the experiments. The experimental procedures were approved by the Institutional Ethical Review Committee and the Italian Ministry of Health (authorization no. 223/2017‐PR) in accordance with the European Union and Italian directives (2010/63EU, D.Lgs 26/2014). The mouse strain was bred at Fondazione Santa Lucia Animal Facility. Mice were housed in groups of 4 per cage in a temperature‐controlled room with 12‐hour light–dark cycle. Water and food were provided ad libitum.

### Electrophysiology

Mice were killed, and their brains were removed as previously described.^30^ Corticostriatal coronal slices (220 μm) were cut with a vibratome in oxygenated artificial cerebrospinal fluid (aCSF). Slices recovered for a minimum of 20 minutes and then were placed in a chamber superfused at 2.5 to 3.5 mL/min with oxygenated aCSF. ChIs were visualized with infrared‐differential interference contrast (IR‐DIC) videomicroscopy and identified based on both their large somatic size and their distinctive electrophysiological properties.^30^, ^31^, ^32^ Electrophysiological signals were detected using Multiclamp 700B, AxoPatch 200, and Axoclamp B amplifiers (Molecular Devices, San Jose, CA, USA). For cell‐attached patch‐clamp recordings, electrodes (4–7 MΩ) were filled with a solution containing 125 mM K‐gluconate, 1 mM CaCl_2_, 2 mM MgCl_2_, 0.1 mM 1,2‐bis‐o‐aminophenoxy‐ethane‐N,N,N′,N′‐tetraacetic acid (BAPTA), 10 mM NaCl, 19 mM HEPES hemisodium salt (*N*‐(2‐hydroxyethyl)‐piperazine‐*N*‐s‐ethanesulfonic acid), 0.3 mM Na‐guanosine triphosphate, and 2 mM Mg‐adenosine triphosphate, pH 7.3. Inhibitory postsynaptic currents (IPSCs) were evoked in ChIs, during whole‐cell patch‐clamp recordings, by intrastriatal paired‐pulse (50‐ms interval) electrical stimulation in the presence of 6‐cyano‐7‐nitroquinoxaline‐2,3‐dione (CNQX, 10 μM) and D‐(‐)‐2‐Amino‐5‐phosphonopentanoic acid (D‐AP5, 20 μM) to avoid glutamate signaling contamination. Parasagittal striatal slices (290 μm) were used for intracellular recordings, with sharp electrodes filled with 2 M KCl (50 MΩ). A bipolar tungsten electrode was positioned in the *corpus callosum* white matter to activate corticostriatal fibers. Glutamatergic excitatory postsynaptic potentials (EPSPs) were evoked by 0.1‐Hz electrical stimulation, in the presence of picrotoxin (50 μM) in the perfusing solution, to block GABAA‐mediated transmission.^30^ High‐frequency stimulation (HFS, three trains: 100 Hz, 3‐second duration, 20‐second interval) was delivered to induce LTD.

### Confocal Imaging

Deeply anesthetized mice were perfused with 4% paraformaldehyde in 0.12 M sodium phosphate buffer (pH 7.5). Brains were postfixed overnight in the same solution and stored at 4°C. Thirty‐micrometer sections were cut using a vibratome (SM 2010R, Leica, Milan, Italy) and maintained in 30% sucrose overnight at 4°C. Then sections were dehydrated with serial alcohol dilutions (50–70–50%) and incubated 1 hour at room temperature in a solution containing 10% donkey serum in phosphate‐buffered saline (PBS) 0.25%‐Triton X‐100 (PBS‐Tx). Finally, they were incubated 3 days at 4°C with the following primary antibodies: goat anti‐ChAT (choline acetyltransferase) (1:500, NBPI30052, Novus Biologicals, Bio‐Techne, Milan, Italy) and rabbit anti‐VAChT (1:500, 139103, Synaptic Systems, Goettingen, Germany). Sections were rinsed thrice for 10 minutes in Tris Buffered Saline (TBS) and incubated at room temperature for 2 hours with 1:200 Alexa 488 or Alexa 647 (Invitrogen, Thermo Fisher Scientific, Milan, Italy). Sections were rinsed for 10 minutes thrice in TBS before mounting with Super Frost Plus (Thermo Scientific, Rodano (MI) Italy). Images were acquired using an LSM700 Zeiss confocal laser scanning microscope (Zeiss, Milan, Italy); confocal images were acquired as previously described.^31^, ^33^

### Immunoblotting

Dorsal striata were homogenized in lysis buffer (50 mM Tris–HCl pH 7.4, 150 mM NaCl, 1% Triton X‐100, 0.25% Na deoxycholate, 5 mM MgCl_2_, 0.1% sodium dodecyl sulfate, and 1 mM EDTA) supplemented with 1% protease inhibitor cocktail (Sigma‐Aldrich, Merck, Milano, Italy). Lysates were sonicated, incubated 1 hour on ice, and then centrifuged at 14,000 rpm for 15 minutes at 4°C. Protein concentration was determined using the Bradford assay (Bio‐Rad, Milan, Italy). NuPAGE LDS sample buffer (Invitrogen, Life Technologies), containing 100 mM 1,4‐dithiothreitol (DTT), was added to the samples. Twenty‐five micrograms of proteins were loaded onto sodium dodecyl sulfate polyacrylamide gel electrophoresis. Then, polyvinylidene fluoride membranes were probed with the following primary antibodies: anti‐torsinA (1:800, ab34540, Abcam, Prodotti Gianni, Milan, Italy), anti‐VGLUT3 (vesicular glutamate transporter 3) (1:000, 135203, Synaptic Systems, Goettingen, Germany), anti‐CHT1 (62‐2ES) (1:500, SC33713, Santa Cruz, D.B.A., Milan, Italy), anti‐ChAT (1:1000, NBP1‐30052, Novus Biologicals, Bio‐Techne, Milan, Italy), anti‐VAChT (1:1000, 139103, Synaptic Systems, Goettingen, Germany), and anti‐β‐actin (1:20,000, A5441, Sigma‐Aldrich). Then, membranes were incubated for 1 hour at room temperature with secondary antibodies. The immunoblot bands were detected with a chemiluminescence system (iBright or film, Thermo Fisher Scientific, Milan, Italy), and their densities were quantified using ImageJ software, National Institutes of Health, USA. For pharmacological treatments with 10 μM sulpiride or 10 μM hemicholinium, 200‐μm thick dorsal striatum slices were incubated for 6 hours at 32°C in oxygenated aCSF and 1% protease inhibitor cocktail (Sigma‐Aldrich). At the end of treatment, the tissue was immediately frozen in liquid nitrogen and stored at −80°C until sample preparation for immunoblotting.

### Gene Expression Analysis by Real‐Time Quantitative Polymerase Chain Reaction

Total RNA was extracted from dorsal striata using TRIreagent (Sigma‐Aldrich, Merck, Milan, Italy) and quantified using an ND‐1000 spectrophotometer (NanoDrop Technologies 2000C, Thermo Scientific, Milan, Italy). RNA integrity was confirmed by 2% agarose gel electrophoresis. Then, 1 mg of total RNA was treated with DNAase I (Invitrogen) and reverse transcribed to complementary cDNA (Roche Italia, Milan, Italy). Real‐time PCR (polymerase chain reaction) was performed on 25 ng of cDNA using specific primers (assay ID 316836 Gene Symbol Slc18a3). Quantitative PCR reactions were carried out in duplicate using the SYBR Green I Master Mix (Roche) on a Roche Light Cycler LC480 system. The relative VAChT gene expression was analyzed using the 2^(–ddCt)^ method.^34^

### Detection of ACh and AChE Activity

For AChE activity assay, mice were killed by cervical dislocation, their brains were removed, and dorsal striata were rapidly dissected at 0°C and stored at –80°C until further analysis. The AChE activity was determined by a colorimetric method, using the AChE assay kit (ab138871, Abcam). The striatal tissue was homogenized in lysis buffer supplemented with 1% protease inhibitor cocktail (Sigma‐Aldrich), with a motor‐driven pestle for three cycles, followed by alternate freezing and thawing of the samples. Dorsal striatum ACh levels were measured using a choline/ACh assay kit (ab65345, Abcam). Absorbance was measured using a Microplate Reader (Multiskan GO, Thermo Scientific). For the measurement of basal ACh content, slices (200–400 μm thick) of dorsal striatum were immediately lysed; in another set of experiments, slices were incubated for 4 minutes at room temperature with 10 μM quinpirole in oxygenated aCSF.

### Drugs

Drugs were bath applied and diluted at the final concentration in aCSF. Vesamicol and neostigmine were obtained from Merck, Milan, Italy. Picrotoxin and donepezil were purchased from Bio‐techne, Milan, Italy.

### Statistical Analysis

Excitatory postsynaptic current (EPSC), Excitatory postsynapticpotential (EPSP), and action potential amplitudes were measured using Clampfit (pClamp 10, Molecular Devices). Statistical analysis was performed using GraphPad Prism. Data were obtained from at least three independent biological samples. All biological replicates are represented by “N,” number of animals, and “n,” number of cells. Values in the text and in the figures are presented as mean ± standard error of the mean. Two‐tailed paired or unpaired *t* test was used for two‐sample comparisons. F test was used to compare dose–response curves. The significance level was set at *P* < 0.05*, *P* < 0.01**, and *P* < 0.001***.

## Results

### VAChT Protein Level Is Increased in the Dorsal Striatum of Tor1a^+/−^ Mice

**FIG. 1:**
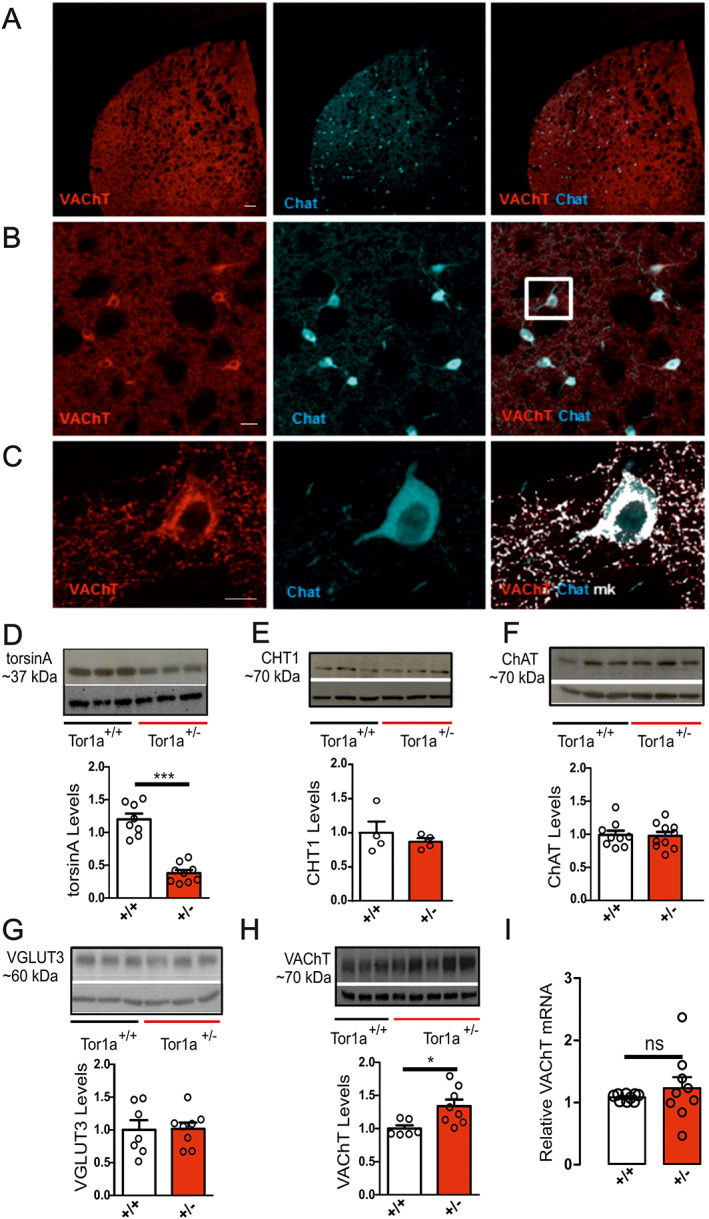
VAChT (vesicular acetylcholine transporter) protein level is increased in the striatum of Tor1a^+/−^ mice. (**A**) Representative confocal fluorescence images of a corticostriatal coronal section confirming cell‐specific expression of VAChT (red) in ChAT (choline acetyltransferase)‐positive cells (cyan) (scale 5×, scale bar 200 μm). Note the absence of immunolabeling in the cortical area. (**B**) Higher‐magnification images showing cholinergic interneurons located in the dorsal striatum (scale 20×, scale bar 50 μm). (**C**) Merged image of the two split channels (ChAT‐cyan and VAChT‐red) and the overlapping mask VAChT/ChAT (mk‐white) to better visualize immunoreactivity of VAChT on ChAT‐positive ChIs (cholinergic interneurons) (scale 63xz1.5, scale bar 10 μm). (**D–H**) Representative Western blots (25 μg of total striatal extract) and the respective densitometry analysis. Histograms show the amount of protein relative to β‐actin (42 kD), used as an internal loading control, and normalized to the wild‐type samples of the same experiment. (**D**) TorsinA (37 kDa) protein level was reduced in the dorsal striatum of Tor1a^+/−^ mice (Tor1a^+/+^ 1.20 ± 0.09, N = 8; Tor1a^+/−^ 0.38 ± 0.05, N = 9; unpaired *t* test *P* < 0.0001***). (**E**) CHT1 (~70 kDa), (**F**) ChAT (~70 kDa), and (**G**) VGLUT3 (vesicular glutamate transporter 3) (~60 kDa) quantification did not show significant changes in Tor1a^+/−^ striatum (CHT1, Tor1a^+/+^ 1.000 ± 0.163, N = 4; Tor1a^+/−^ 0.868 ± 0.053, N = 4, unpaired *t* test *P* = 0.47; ChAT, Tor1a^+/+^ 0.99 ± 0.06, N = 9; Tor1a^+/−^ 0.97 ± 0.06, N = 10; unpaired *t* test *P* = 0.85; VGLUT3, Tor1a^+/+^ 1.00 ± 0.15, N = 7; Tor1a^+/−^ 1.01 ± 0.10, N = 8; unpaired *t* test *P* = 0.93). (**H**) VAChT (~70 kDa) densitometry analysis revealed a significant increase in protein level in Tor1a^+/−^ mice (Tor1a^+/+^ 1.00 ± 0.05, N = 6; Tor1a^+/−^ 1.33 ± 0.10, N = 8; unpaired *t* test *P* = 0.0176*). Data are presented as mean ± standard error of the mean (SEM). (**I**) VAChT mRNA copy numbers in Tor1a^+/−^ and Tor1a^+/+^ striatal samples were determined by quantitative real‐time polymerase chain reaction. The dot‐plot graph shows the relative expression of VAChT mRNA normalized to the expression of the reference gene Hprt1 for each sample. Tor1a^+/−^ values are presented as fold change (2^(–ddCt)^) with respect to Tor1a^+/+^ samples. No statistically significant difference was found between genotypes (1.23 ± 0.18 fold change, N = 9, unpaired *t* test *P* = 0.23). Data are presented as mean ± SEM. [Color figure can be viewed at wileyonlinelibrary.com]

A functional cholinergic neurotransmission is maintained through an appropriate synthesis, vesicular packaging, and release of ACh. Our confocal microscope analysis showed that VAChT is localized to the soma of large‐sized ChIs in Tor1a^+/−^ striatum, similar to wild‐type mice (not shown) as well as to the known distribution of the ACh synthesizing enzyme ChAT (Fig. 1A–C). In addition, VAChT immunostaining was abundantly present in axonal varicosities, as punctiform labeling (Fig. 1B,C). Indeed, high‐magnification images show coexpression of VAChT and ChAT in ChIs, consistent with previous observations.^35^, ^36^

We then evaluated CHT1, ChAT, and VAChT protein expression levels in the dorsal striatum. As previously reported, torsinA protein level was reduced in Tor1a^+/−^ mice compared to controls (Fig. 1D; *P* < 0.0001***). CHT1 (Fig. 1E) and ChAT (Fig. 1F) protein levels were not significantly different in Tor1a^+/+^ versus Tor1a^+/−^ striatum (CHT1, *P* = 0.47; ChAT, *P* = 0.85). Striatal ChIs coexpress VGLUT3.^37^, ^38^ VGLUT3 protein level was similar in the striatum of wild‐type and mutant mice (Fig. 1G; VGLUT3, *P* = 0.93). VAChT immunoreactivity revealed an intense band at the predicted size of 70 kDa (Fig. 1H). Quantification of band intensity revealed a significant increase in VAChT protein level in Tor1a^+/−^ mice, as compared to Tor1a^+/+^ control mice (Fig. 1H; *P* = 0.0176*). Therefore, we verified whether the increase in VAChT protein abundance was caused by an enhancement in mRNA level. However, we found no difference in the expression of VAChT (*Slc18a3*) mRNA between Tor1a^+/−^ and Tor1a^+/+^ mice (Fig. 1I; *P* = 0.23). Our findings provide evidence for a selective increase in VAChT protein level in the dorsal striatum of Tor1a^+/−^ in spite of no significant changes in mRNA expression.

### ACh Content Is Elevated in the Striatum of Tor1a^+/−^ Mice Both in Basal Condition and after D2R Activation

**FIG. 2:**
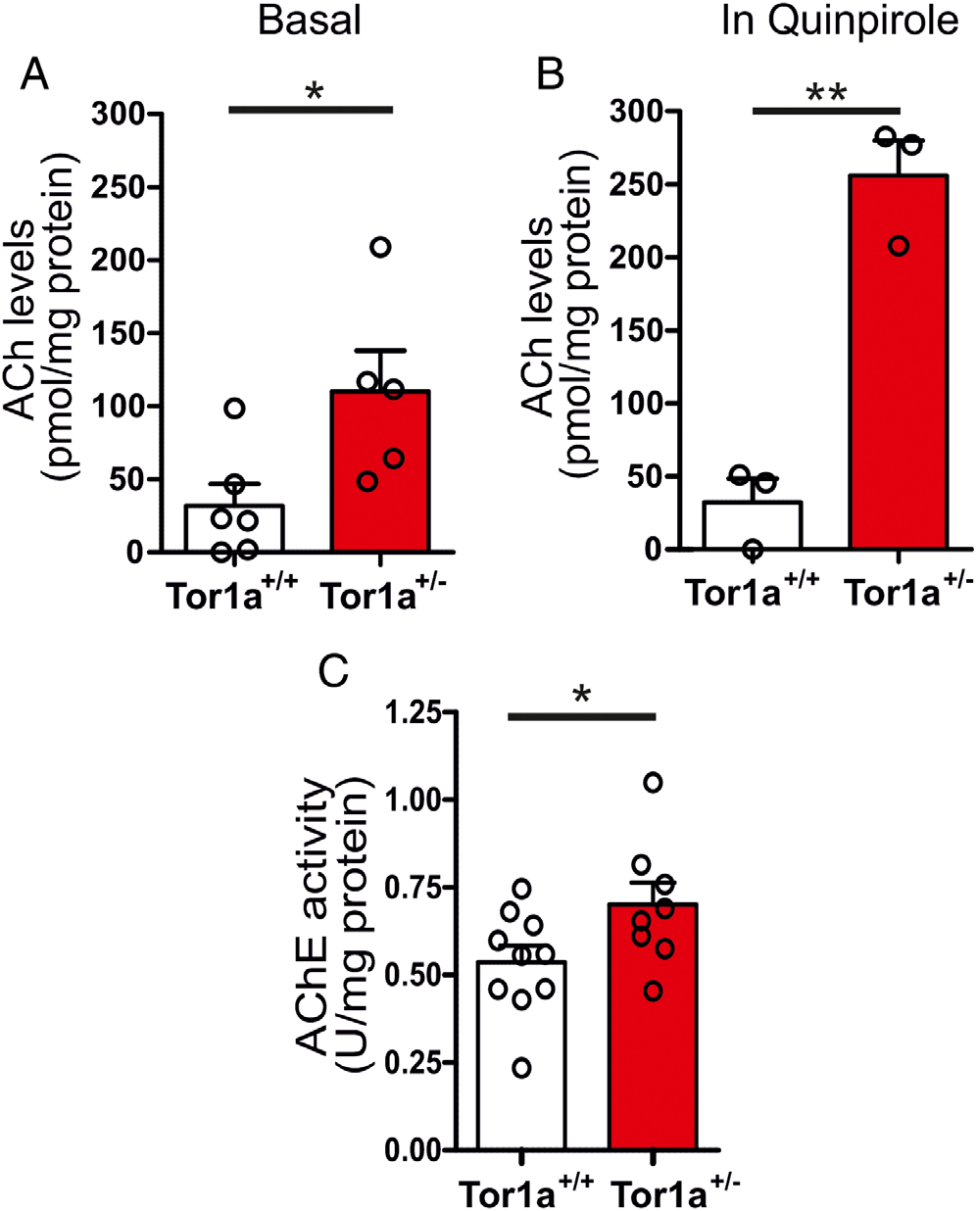
Increased striatal ACh (acetylcholine) content and AChE (acetylcholinesterase) activity in Tor1a^+/−^ mice. VAChT (vesicular acetylcholine transporter) protein expression in vitro after ACh system modulation. (**A**) The histogram shows an enhanced basal ACh content in mutant mice (Tor1a^+/+^ 31.88 ± 15.01 pmol/mg protein, N = 6; Tor1a^+/−^110.1 ± 27.99 pmol/mg protein, N = 5; unpaired *t* test *P* = 0.0293*). (**B**) Quinpirole (10 μM, 4 minutes) dramatically increases striatal ACh content in Tor1a^+/−^, as compared to control, mice (Tor1a^+/+^ 32.34 ± 16.24 pmol/mg protein; N = 3; Tor1a^+/−^ 255.8 ± 24 pmol/mg protein in N = 3; unpaired *t* test *P* = 0.001**). (**C**) The plot shows a significant increase in AChE activity in the dorsal striatum of Tor1a^+/−^ mice (Tor1a^+/+^ 0.54 ± 0.05 U/mg total proteins, N = 10; Tor1a^+/−^ 0.70 ± 0.06 U/mg total proteins, N = 8; unpaired *t* test *P* = 0.048*). [Color figure can be viewed at wileyonlinelibrary.com]

These findings prompted us to measure ACh content in striatal slices from Tor1a^+/−^ mice. To minimize ACh degradation, we quickly collected the samples in liquid nitrogen. By means of a colorimetric assay, we measured an increase in striatal ACh content in Tor1a^+/−^ with respect to wild‐type mice (Fig. 2A; *P* = 0.0293*). We previously found that the activation of D2R increases the frequency of ChI spontaneous firing activity in Tor1a^+/−^ mice,^19^ similar to that reported in multiple DYT1 models.^12^, ^18^, ^21^, ^32^, ^39^ To verify that D2R‐dependent increase in firing frequency would result in an elevation in ACh release in Tor1a^+/−^ mice, we measured ACh content after striatal slice treatment with the D2R agonist quinpirole. Bath application of quinpirole (10 μM, 4 minutes) did not significantly affect ACh content in wild‐type slices, whereas it increased striatal ACh level in Tor1a^+/−^ striatum (Fig. 2B; *P* = 0.001**). Overall, these data show that basal ACh content is enhanced in the striatum of Tor1a^+/−^ mice, in line with microdialysis experiments performed in Tor1a^+/Δgag^ mice.^12^ Furthermore, our experiments demonstrate that D2R activation abnormally elevates ACh tone in Tor1a^+/−^ mice.

To investigate whether the abnormal D2R activity might cause the increase in VAChT protein level observed in Tor1a^+/−^ mice, we treated dorsal striatum slices with the D2R antagonist sulpiride (10 μM, 6 hours, 32°C). This prolonged treatment did not alter VAChT protein level with respect to untreated, contralateral slices (Fig. S1A; Tor1a^+/+^ *P* = 0.433; Tor1a^+/−^ *P* = 0.431). There was no statistically significant difference in the effect of sulpiride on VAChT protein expression between genotypes (*P* = 0.94). To further rule out the role of the enhanced striatal ACh tone in the increase in VAChT, we performed a treatment of slices with hemicholinium (10 μM, 6 hours, 32°C). Similar to sulpiride, hemicholinium did not alter VAChT protein level with respect to untreated, contralateral slices (Fig. S1B; Tor1a^+/+^ *P* = 0.40, Tor1a^+/−^ *P* = 0.81). There was no statistically significant difference in the effect of the treatment with hemicholinium between genotypes (*P* = 0.63).

### AChE Activity Is Increased in Tor1a^+/−^ Mice

AChE is the enzyme that catalyzes the rapid breakdown of ACh in the striatum.^40^, ^41^ By means of a colorimetric assay, we therefore measured the enzymatic activity and found a significant increase in AChE activity in Tor1a^+/−^ compared to wild‐type mice (Fig. 2C; *P* = 0.048*). These results indicate that the increased rate of enzymatic degradation is likely a compensatory mechanism triggered by the elevation of basal ACh levels.

### Reduced Potency of AChE Inhibitors on Spontaneous Firing Activity of Tor1a^+/−^ ChIs

**FIG. 3:**
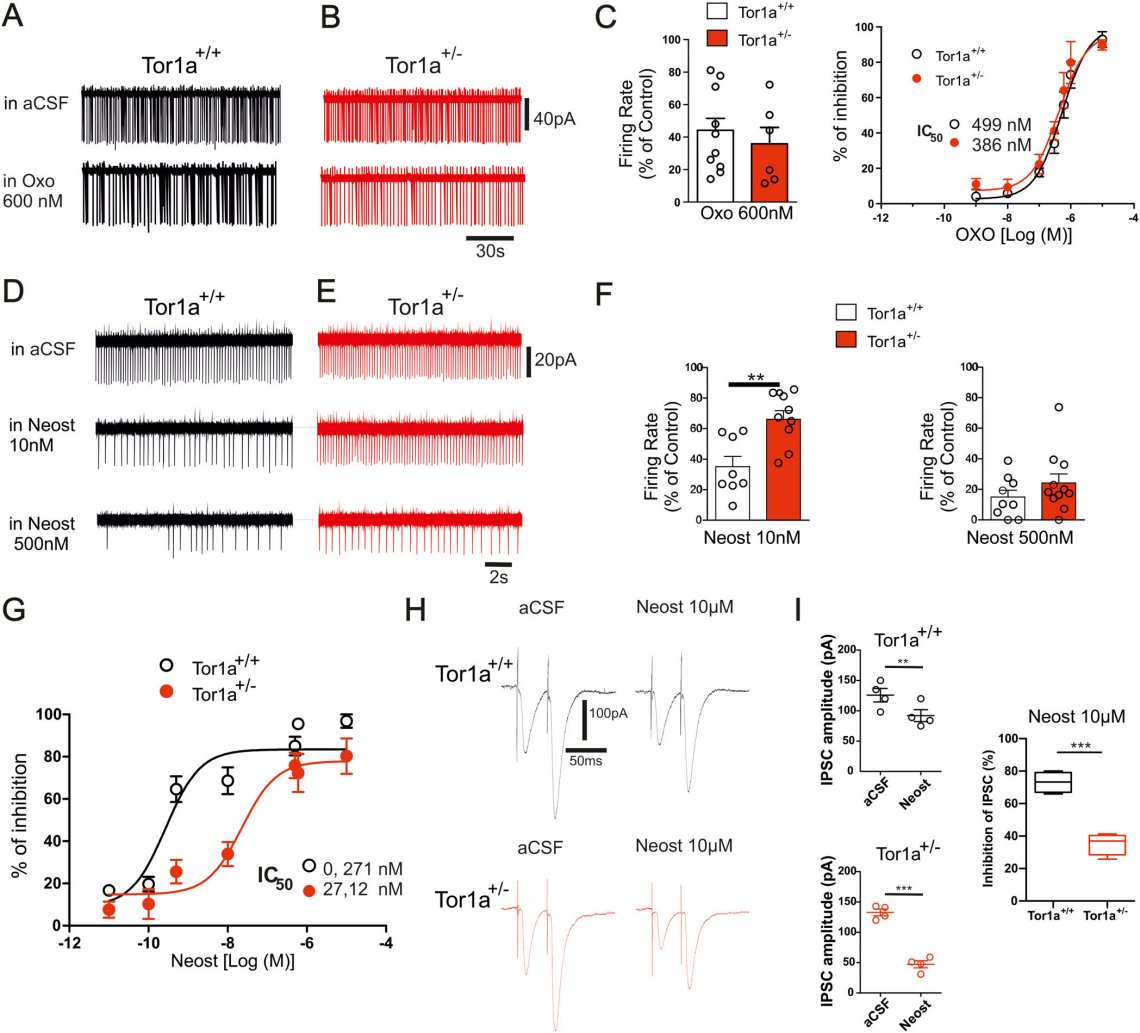
Reduced potency of AChE (acetylcholinesterase) inhibitors on the spontaneous firing activity of Tor1a^+/−^ ChIs (cholinergic interneurons). (**A, B**) Representative patch‐clamp recordings of ChIs in aCSF (artificial cerebrospinal fluid) (top) and after bath application of oxotremorine (Oxo 600 nM, 2 minutes, bottom) in both strains. (**C**) (Left) Summary plot showing a similar decrease in firing rate induced by 600 nM Oxo in both genotypes (Tor1a^+/+^: 44.15 ± 7.35% of control; n = 11, N = 5; Tor1a^+/−^: 35.85 ± 10.07% of control; n = 6, N = 3; unpaired *t* test *P* = 0.51). (Right) The dose–response curves show similar kinetics of inhibition in both genotypes (IC50: Tor1a^+/+^ 499 nM, 95% confidence intervals: 3.463e‐007 to 7.212e‐007; Tor1a^+/−^ 386 nM, 2.522e‐007 to 5.932e‐007; F test *P* = 0.51). (**D, E**) Representative traces of cell‐attached recordings from ChIs in aCSF (top) and after bath application of 10 nM (middle) and 500 nM (bottom) neostigmine (Neost, 5 minutes). (**F**) The histograms summarize the effect of 10 nM (left, Tor1a^+/+^ 35.11 ± 6.71% of control, n = 8, N = 6; Tor1a^+/−^ 66.07 ± 5.69% of control, n = 9, N = 7; unpaired *t* test *P* = 0.0034**) and 500 nM of Neost (right, Tor1a^+/+^ 14.98 ± 4.44% of control; n = 9, N = 6; Tor1a^+/−^ 24.13 ± 6.01% of control; n = 11, N = 8; unpaired *t* test *P* = 0.25). (**G**) The dose–response curves of Neost inhibitory effect on ChI firing activity show a shift to the right in Tor1a^+/−^ compared to wild‐type cells (Tor1a^+/+^: IC50 0.271 nM, 1.119e‐010 to 6.598e‐010 confidence interval; Tor1a^+/−^: IC50 27.12 nM, 6.797e‐009 to 1.082e‐007 95% confidence interval; F test *P* < 0.0001***). **(H)** Representative traces of IPSCs (inhibitory postsynaptic currents) evoked in ChIs by paired pulse intrastriatal stimulation (50‐ms interval) in the presence of CNQX (10 μM) and D‐AP5 (20 μM), to avoid glutamate signaling contamination, before (left) and after (right) bath application of Neost (10 μM, 15 minutes). (**I)** The graphs summarize the effect of Neost on the IPSC amplitude (Tor1a^+/+^: in aCSF 125.8 ± 12 pA, in Neost 92 ± 10 pA, n = 4, paired *t* test *P* = 0.0054**; Tor1a^+/−^: in aCSF 132.8 ± 6 pA, in Neost 47.3 ± 6 pA, n = 4, paired *t* test *P* = 0.019**). The extent of IPSC amplitude reduction is significantly higher in Tor1a^+/−^ mice than in Tor1a^+/+^ mice (Tor1a^+/+^ 73.11 ± 3.17% of control, n = 4; Tor1a^+/−^ 35.26 ± 3.32% of control, n = 4; unpaired *t* test *P* = 0.0002***). Data are presented as mean ± standard error of the mean. [Color figure can be viewed at wileyonlinelibrary.com]

ACh exerts an inhibitory feedback control on ChIs activity through the activation of M2/M4 mACh autoreceptors.^42^, ^43^, ^44^ To investigate whether the enhancement of striatal ACh content could be caused by an altered autoreceptor function, we carried out an electrophysiology analysis of ChI excitability, measuring the responses to autoreceptor agonists. All the recorded neurons exhibited typical morphological features, such as a large polygonal body and two to three dendritic branches, as well as characteristic electrophysiological properties. Basal spontaneous firing rates did not statistically differ between genotypes (Tor1a^+/+^ 2.76 ± 0.43 Hz, n = 34, N = 23; Tor1a^+/−^ 3.11 ± 0.40 Hz, n = 27, N = 25; unpaired *t* test, *P* = 0.56). Bath application of the M2/M4 mAChR agonist oxotremorine (Fig. 3A,B, OXO 600 nM, 2 minutes) induced a decrease in ChI spontaneous firing rate in both TorA^+/+^ and TorA^+/−^ mice (Fig. 3C; *P* = 0.51). A dose–response curve showed that OXO acts with the same efficacy in both genotypes (Fig. 3C; *P* = 0.51).

AChE inhibitors enhance striatal ACh level, which tonically activates mACh autoreceptors on ChIs. As expected, bath application of neostigmine (Neost, 10 nM, 5 minutes) produced a decrease in the number of spontaneous action potentials in Tor1a^+/+^ mice (Fig. 3D,F). Remarkably, Neost was less effective in reducing the average firing rate in Tor1a^+/−^ slices (Fig. 3E,F; *P* = 0.0034). Similarly, donepezil, a reversible AChE inhibitor, produced a transient decrease in the number of spontaneous action potentials in Tor1a^+/+^ mice (Fig. S2A,B; *P* = 0.0416*).

Pretreatment with the nonselective mAChR antagonist scopolamine (3 μM, 20 minutes) abolished the inhibitory effect of neostigmine (10 nM, 5 minutes) (data not shown). The inhibitory effect of a higher concentration of Neost on the firing rate (500 nM; Fig. 3D–F) was similar between genotypes (Fig. 3F; *P* = 0.25). As shown by the dose–response analysis (Fig. 3G), Neost‐induced effects on firing rate were significantly different between Tor1a^+/−^ and Tor1a^+/+^ ChIs. The dose–response curve of Tor1a^+/−^ mice was shifted to the right with an IC50 value significantly higher with respect to Tor1a^+/+^ (Fig. 3G; *P* < 0.0001***), demonstrating that Neost acts with the same efficacy in both genotypes but with a lower potency in Tor1a^+/−^.

Muscarinic M1 receptors were shown to modulate inhibitory inputs from GABAergic terminals targeting ChIs.^45^ Thus, to further evaluate the effect of an abnormal ACh level on striatal circuitry, we investigated the modulatory role of ACh on the GABAergic transmission from striatal projection neurons and parvalbumin‐expressing interneurons onto ChIs in both animal groups. IPSCs were evoked in ChIs by intrastriatal stimulation. In both Tor1a^+/+^ and Tor1a^+/−^ mice, the increase in intrastriatal ACh induced by bath application of neostigmine (10 μM, 15 minutes) caused a significant decrease in IPSC amplitude (Fig. 3H; Tor1a^+/+^ *P* = 0.0054**; Tor1a^+/−^ *P* = 0.019**), which was significantly higher in Tor1a^+/−^ than in Tor1a^+/+^ mice (Fig. 3I; *P* = 0.0002***), further supporting an increased ACh tone in DYT1 mice.

Altogether, our data indicate that mACh autoreceptor function is preserved in Tor1a^+/−^ mice and suggest that AChE inhibition is less effective in modulating ChI firing activity because of the stable increase in enzymatic activity in Tor1a^+/−^ mice.

### Inhibition of VAChT Restores Striatal Long‐Term Depression

**FIG. 4:**
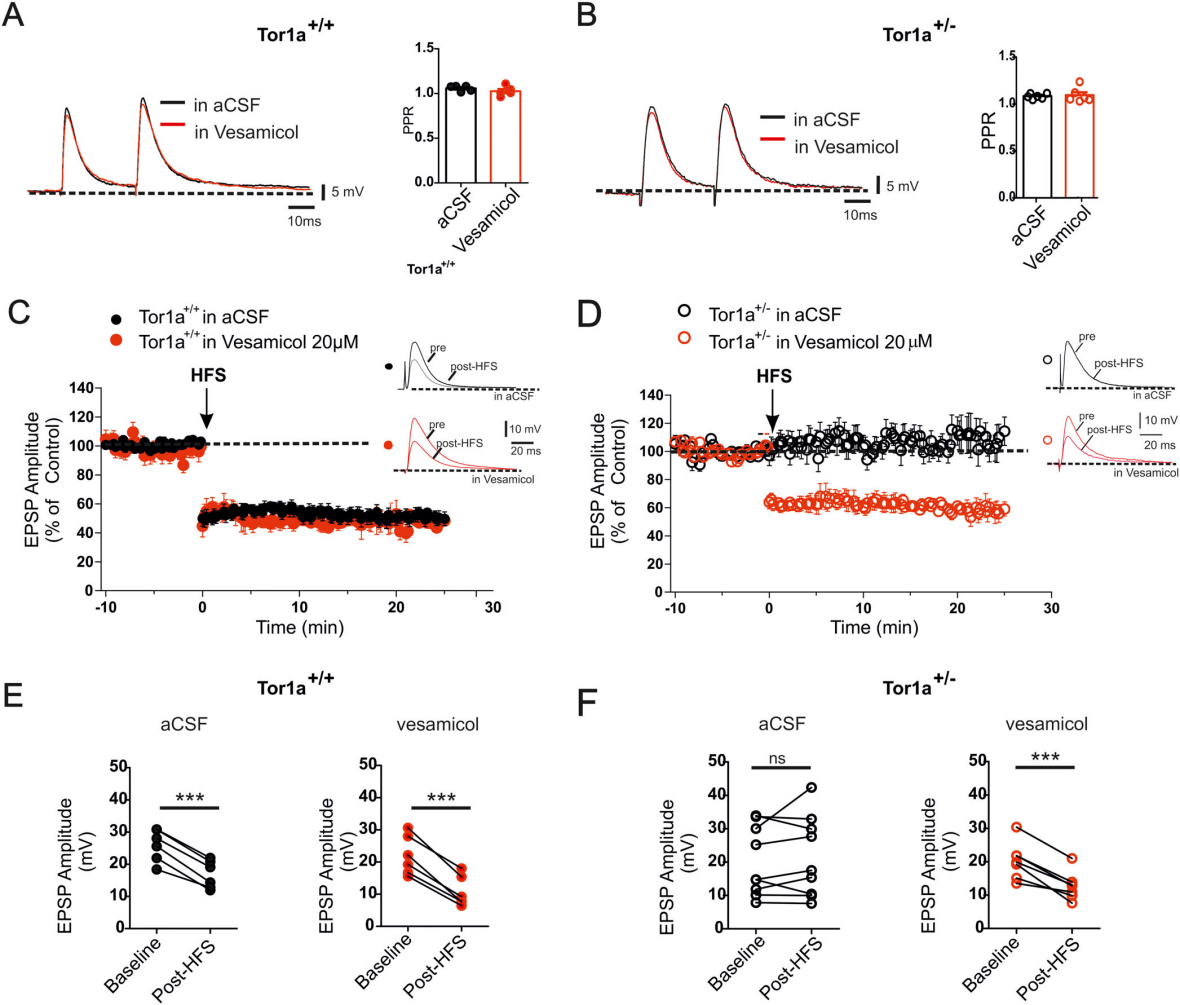
Inhibition of VAChT (vesicular acetylcholine transporter) rescues LTD (long‐term depression) in Tor1a^+/−^ mice. (**A**) Slice perfusion with the VAChT‐blocking agent vesamicol (20 μM, 20 minutes) does not modify the paired‐pulse ratio (PPR) of corticostriatal EPSPs (excitatory postsynaptic potentials) in Tor1a^+/+^ MSNs, as shown by (left) the representative traces of intracellular recordings and (right) the summary plot (in aCSF [artificial cerebrospinal fluid] 1.06 ± 0.01, n = 5, N = 4; in vesamicol 1.03 ± 0.03, n = 5, N = 4; paired *t* test *P* = 0.268). (**B**) Vesamicol does not modify the PPR of corticostriatal EPSPs in Tor1a^+/−^ MSNs: (left) representative traces of intracellular recordings and (right) summary plot (in aCSF 1.08 ± 0.01, n = 6, N = 4; in vesamicol 1.09 ± 0.03, n = 6, N = 4; paired *t* test *P* = 0.682). (**C**) Time course of corticostriatal LTD induced by HFS (high‐frequency stimulation) (black arrow) in Tor1a^+/+^ MSN, either in aCSF (black circles) or in 20‐μM vesamicol (red circles). (**D**) Time course of corticostriatal LTD, induced by HFS (black arrow) in Tor1a^+/−^ MSN, either in aCSF (black circles) or in 20‐μM vesamicol (red circles). (**E**) The amplitude of Tor1a^+/+^ corticostriatal LTD was similar in aCSF and in vesamicol (aCSF, black circles: pre‐HFS 25.92 ± 2.04 mV; post‐HFS 16.82 ± 1.76 mV; n = 6, N = 4; paired *t* test *P* < 0.0001***; vesamicol, red circles: pre‐HFS: 22.05 ± 2.50; post‐HFS 10.73 ± 1.95 mV; n = 6, N = 5; paired *t* test *P* = 0.0002***). (**F**) HFS did not induce a corticostriatal LTD in Tor1a^+/−^ MSNs (aCSF, black circles: pre‐HFS 20.23 ± 3.48 mV; post‐HFS 21.53 ± 4.04 mV; *n* = 9, N = 5; paired *t* test *P* = 0.4555). Vesamicol preincubation was able to rescue LTD expression in the striatum of Tor1a^+/−^ mice (pre‐HFS 20.25 ± 2.07 mV; post‐HFS 12.74 ± 1.60 mV; n = 7, N = 5; paired *t* test *P* = 0.0005***). [Color figure can be viewed at wileyonlinelibrary.com]

A prominent involvement of cholinergic transmission in the impairment of striatal synaptic plasticity has been shown in DYT1 dystonia models, where a loss of corticostriatal LTD has been reported.^20^, ^21^, ^23^, ^24^, ^25^ We investigated whether, by blocking VAChT and therefore reducing ACh uptake into synaptic vesicles and, in turn, ACh release, we might rescue corticostriatal LTD. EPSPs were evoked by 0.1‐Hz stimulation of corticostriatal fibers. Slice perfusion with vesamicol (20 μM, 20 minutes), a VAChT‐blocking agent, did not modify the intrinsic membrane properties of medium spiny neurons (MSNs) (not shown), as well as the amplitude and paired‐pulse ratio (PPR) of corticostriatal EPSPs in both genotypes (Fig. 4A,B; Tor1a^+/+^ *P* = 0.268; Tor1a^+/−^ *P* = 0.682).

As previously shown in multiple DYT1 rodent models,^20^, ^21^, ^22^, ^23^, ^24^, ^25^, ^30^ delivery of HFS induced an LTD in wild‐type but not in Tor1a^+/−^ MSNs (Fig. 4C,D, Tor1a^+/+^ in aCSF: *P* < 0.0001***; Tor1a^+/−^ in aCSF: paired *t* test *P* = 0.4555). Of note, we found that 20‐μM vesamicol was able to rescue the expression of a robust synaptic depression in Tor1a^+/−^ slices (Fig. 4D; Tor1a^+/−^ in vesamicol: *P* = 0.0005***), without affecting LTD in wild‐type neurons (Fig. 4C; Tor1a^+/+^ in vesamicol: *P* = 0.0002***).

**FIG. 5:**
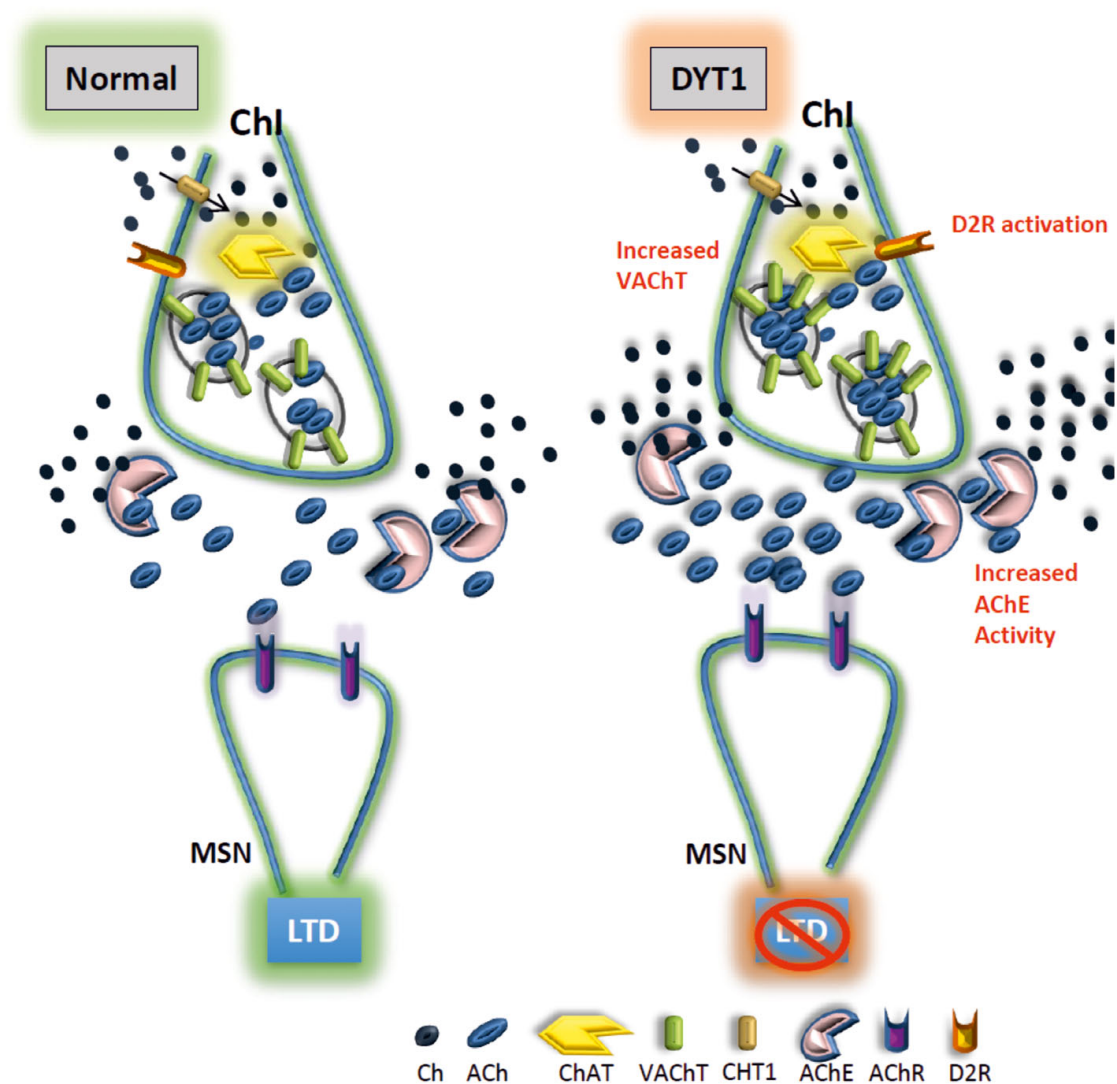
Simplified model of cholinergic synaptic dysfunction in DYT1 dystonia. (Left) Cartoon showing normal signaling between ChIs (cholinergic interneurons) and MSNs, leading to the expression of normal LTD (long‐term depression). In DYT1 dystonia (right), synaptic vesicles contain more VAChT (vesicular acetylcholine transporter), which allows to store significant amounts of ACh. During LTD induction, activation of an abnormal D2R (dopamine 2 receptor) increases the release of these vesicles fully loaded with ACh into the synaptic cleft. Despite the increase in AChE (acetylcholinesterase) activity, which helps degrading ACh, the excessive cholinergic tone disrupts the expression of synaptic plasticity in MSNs. [Color figure can be viewed at wileyonlinelibrary.com]

These data support the hypothesis that an increased presynaptic VAChT protein level elevates the vesicular packaging of ACh, thus allowing an enhanced release in the dorsal striatum and generating an increased ACh tone, which prevents LTD induction (Fig. 5).

## Discussion

The paucity of novel drugs for the treatment of dystonia is determined by the lack of definite therapeutic targets. Although dystonia is not typically associated with degeneration or obvious neuropathological features, striatal dysfunction, including abnormal cholinergic transmission, is consistently implicated in multiple forms of dystonia, including DYT1 dystonia. The aim of the present study was to better define the mechanistic basis for such abnormality in the DYT1 striatum. Our results indicate that the striatal cholinergic machinery is altered at multiple levels in Tor1a^+/−^ mice, ultimately producing a major elevation in ACh content.

ACh is synthesized by ChAT from the substrates choline (taken up into the cell by the high‐affinity choline transporter CHT1) and acetylCoA. ACh is then packaged into vesicles by VAChT, which is encoded by a small gene embedded in the gene‐producing ChAT, and its action is terminated by AChE. Although we found no significant differences in ChAT or CHT1 protein expression, we measured an increase in VAChT protein amounts in mutant mice, without changes in mRNA levels. In fact, protein abundance is regulated at different levels, including protein degradation.^46^ The finding of an increased level of the transporter despite the fact that the synthesizing enzyme is unaffected is not surprising. Previous studies found that an increased protein level of VAChT does not imply the involvement of other cholinergic markers.^36^, ^47^, ^48^

Given the critical role of VAChT in cholinergic signaling, as a rate‐limiting factor for replenishing cholinergic vesicles, our data show that an increased VAChT activity may be able to sustain enhanced ACh storage and release from synaptic vesicles in Tor1a^+/−^ mice. These findings are in agreement with previous observations showing that a similar increase in VAChT protein level leads to an increase in cholinergic tone.^48^, ^49^ Accordingly, transgenic VAChT‐deficient mice show decreased basal and evoked ACh release in the striatum.^50^ Furthermore, VAChT overexpression induces changes in the morphology of striatal ChIs.^36^ Interestingly, these observations are consistent with a previous work reporting an enlargement of striatal ChIs in a knock‐in mouse model of DYT1 dystonia.^51^

Spontaneous firing activity of ChIs ensures a basal cholinergic tone in the striatum,^52^, ^53^, ^54^ which is negatively modulated by M2/M4 muscarinic autoreceptors and D2R dopamine receptors. Basal firing frequency was not altered in Tor1a^+/−^ ChIs, and the muscarinic autoreceptor response was conserved, as previously reported.^12^, ^19^, ^55^ AChE inhibition, by increasing endogenous ACh content, caused a decrease in ChI spontaneous firing activity in both wild‐type and mutant striatal slices. However, the decrease in firing rate in Tor1a^+/−^ ChIs was less pronounced compared to Tor1a^+/+^. This observation is in accordance with present data showing an increased AChE activity in Tor1a^+/−^ striatum and in line with previous reports from a different DYT1 mouse model.^20^ We hypothesize that the lack of changes in ChIs basal firing frequency, despite an increased ACh tone, might be due to the overactivity of AChE, preventing an increased activation of muscarinic autoreceptors. Such an increase in AChE activity may represent a compensatory mechanism to limit the abundance of ACh in the synaptic cleft. The physiological relevance of the increased ACh tone was demonstrated by our observation that, on AChE maximal inhibition, the amplitude of GABAergic synaptic currents was decreased more in Tor1a^+/−^ than in wild‐type ChIs.

Similar to M2/M4 autoreceptors, in physiological conditions also D2R activation exerts an inhibitory action on striatal ChI firing activity. However, in several DYT1 rodent models we and others reported that the activation of D2R causes a significant increase in ChI firing rate.^12^, ^18^, ^19^ Here we demonstrate that the activation of striatal D2R produces a significant increase in ACh content in striatal slices of Tor1a^+/−^ mice. Indeed, a “paradoxical excitation” of ChIs induced by the activation of D2R has been reported also in DYT6 and DYT25 mouse models of genetic dystonia.^56^

In the striatum, dopaminergic and cholinergic signaling act synergistically and reciprocally to shape synaptic plasticity. In the Tor1a^+/−^ mouse model, we previously reported the loss of LTD and suggested that this impairment could result from a cholinergic overactivity, because it can be reverted by lowering ACh content or inhibiting postsynaptic muscarinic M1 receptors.^20^ In support of this hypothesis, the present data show that VAChT inhibition by vesamicol was able to rescue LTD expression in Tor1a^+/−^ MSNs. Indeed, an increased level of VAChT protein allows either synaptic vesicles to be filled with more ACh or more vesicles to be filled and released, therefore supporting a sustained release under HFS. Because vesamicol did not change corticostriatal PPR, we hypothesize that VAChT inhibition, by limiting ACh release, may dampen the activation of postsynaptic M1 receptors located on MSNs, thus rescuing LTD expression, in accordance with our previous work.^25^ Indeed, though the anticholinergic drug trihexyphenidyl, currently used in the management of dystonia, does not display selectivity for M1 receptors,^57^ we showed that only M1‐selective and M1‐preferring antagonists were able to offset synaptic plasticity deficits in DYT1 mice.^25^

The mechanisms by which mutant torsinA leads to the specific modification of cholinergic transmission with an increased protein level of VAChT and long‐lasting alterations in circuit function are still unknown. The high expression of torsinA in striatal ChIs might explain a preferential vulnerability of these neurons.^11^, ^58^ Recent studies suggested a relationship between the loss of ChIs and the expression of a dystonia phenotype in torsinA conditional knockout models.^59^, ^60^ However, there is little evidence supporting the degeneration of cholinergic neurons in DYT1 dystonia patients.^59^ Histological studies in DYT1 patients' striata indicate a preservation of large aspiny interneurons, which are believed to correspond to ChIs.^61^, ^62^, ^63^

Novel, selective VAChT Positron emission tomography (PET) ligands have been recently developed to measure cholinergic function in a number of conditions, including Parkinson's and Alzheimer's diseases.^64^, ^65^ VAChT represents a promising target to measure cholinergic deficits in dystonia patients.^66^, ^67^

Overall, our findings provide a direct demonstration of a significant alteration of the cholinergic transmission in the striatum of DYT1 mice, further adding to substantial evidence in support of the central role of ACh in DYT1 dystonia pathophysiology.

## Author Roles

(1) Research Project: A. Conception, B. Organization, C. Execution; (2) Statistical analysis: A. Design, B. Execution, C. Review and critique; (3) Manuscript preparation: A. Writing of the first draft, B. Review and critique.

A.T.: 1A, 1B, 1C, 2A, 2B, 2C, 3A

G.M.: 1B, 1C, 2A, 2B

M.M.: 1C, 2B

V.V.: 1C, 2B

G.S.: 1C, 3B

G.P.: 1C, 3B

P.I.: 2B, 2C

P.B.: 1B, 2A, 2C, 3B

A.P.: 1A, 1B, 2A, 2C, 3A, 3B

## Financial Disclosures

A.P. is employee at the University of Pavia, Pavia, Italy; P.B. and P.I. are employees at Fondazione Santa Lucia, Rome, Italy; A.P. holds grants that are not related to the subject of the present study. A.T., G.M., M.M., V.V., G.S., G.P., P.I., P.B., A.P. declare the absence of any potential conflict of interest.

## Supporting information


**Figure S1.** (**A**) Left: representative immunoblotting showing VAChT protein expression levels after treatment of striatal slices with either aCSF or 10 μM sulpiride (6 hours, 32°C). Middle: densitometry analysis revealed no significant effects of sulpiride treatment on VAChT protein level, with respect to contralateral, untreated slices, either in Tor1a^+/+^ or Tor1a^+/−^ mice (Tor1a^+/+^: aCSF 2.59 ± 0.32, N = 7; sulpiride 3.03 ± 0.56, N = 7; paired *t* test *P* = 0.433; Tor1a^+/−^: aCSF, 2.74 ± 0.54, N = 6; sulpiride 3.16 ± 0.60, N = 6; paired *t* test *P* = 0.431). Right: summary plot showing no statistically significant difference in the effect of sulpiride treatment between Tor1a^+/+^ and Tor1a^+/−^ genotypes (Tor1a^+/+^: 1.20 ± 0.21, N = 7; Tor1a^+/−^: 1.18 ± 0.18, N = 6; unpaired *t* test *P* = 0.94). (**B**) Similarly, a 6‐hour treatment with 10 μM hemicholinium did not alter VAChT protein level, with respect to contralateral, untreated slices, either in Tor1a^+/+^ or in Tor1a^+/−^ mice, as shown by the representative immunoblotting (left) and the summary plot (middle, Tor1a^+/+^: aCSF 1.73 ± 0.20, N = 6, hemicholinium 1.57 ± 0.12, N = 6, paired *t* test *P* = 0.40; Tor1a^+/−^: aCSF 1.77 ± 0.11, N = 7, hemicholinium 1.88 ± 0.32, N = 6, paired *t* test *P* = 0.81). Right: the plot shows the absence of significant changes in VAChT levels after hemicholinium treatment between Tor1a^+/+^ and Tor1a^+/−^ slices (Tor1a^+/+^: 0.95 ± 0.10, N = 6; Tor1a^+/−^: 1.05 ± 0.17, N = 6; unpaired *t* test *P* = 0.63). Data are presented as mean ± standard error of the mean.

**Figure S2.** (**A, B**) Representative patch‐clamp recordings of ChIs, in aCSF (top) and after bath application of donepezil (Donep 50 μM, 5 minutes, bottom), showing an inhibitory effect on spontaneous firing rate. (**C**) (Left) The inhibition by donepezil was weaker in Tor1a^+/−^ (Tor1a^+/−^ average firing rate: in aCSF 3.59 ± 1.18 Hz; in donepezil 3.30 ± 1.09 Hz; n = 4, N = 3; paired *t* test *P* = 0.5791) than in control slices (Tor1a^+/+^ average firing rate: in aCSF 4.23 ± 1.09 Hz; in donepezil 1.44 ± 0.65 Hz; n = 4, N = 3; paired *t* test 0.0416*). (Right) The histogram summarizes the difference between genotypes (Tor1a^+/+^ 30.79 ± 9.01% of control; n = 4, N = 3; Tor1a^+/−^ 93.46 ± 11.86% of control; n = 4, N = 3; unpaired *t* test *P* = 0.0056**).

## Data Availability

Data available on request from the authors.

## References

[1] Fahn S , Eldridge R . Definition of dystonia and classification of the dystonic states. Adv Neurol 1976;14:1–5. 941763

[2] Ozelius LJ , Hewett JW , Page CE , et al. The early‐onset torsion dystonia gene (DYT1) encodes an ATP‐binding protein. Nat Genet 1997;17(1):40–48. 9288096 10.1038/ng0997-40

[3] Granata A , Koo SJ , Haucke V , et al. CSN complex controls the stability of selected synaptic proteins via a torsinA‐dependent process. EMBO J 2011;30(1):181–193. 21102408 10.1038/emboj.2010.285 PMC3020110

[4] Grillet M , Dominguez Gonzalez B , Sicart A , et al. Torsins are essential regulators of cellular lipid metabolism. Dev Cell 2016;38(3):235–247. 27453503 10.1016/j.devcel.2016.06.017

[5] Rose AE , Brown RSH , Schlieker C . Torsins: not your typical AAA+ ATPases. Crit Rev Biochem Mol Biol 2015;50(6):532–549. 26592310 10.3109/10409238.2015.1091804 PMC4872298

[6] Laudermilch E , Schlieker C . Torsin ATPases: structural insights and functional perspectives. Curr Opin Cell Biol 2016;40:1–7. 26803745 10.1016/j.ceb.2016.01.001 PMC4887320

[7] Gonzalez‐Alegre P . Advances in molecular and cell biology of dystonia: focus on torsinA. Neurobiol Dis 2019;127:233–241. 30877032 10.1016/j.nbd.2019.03.007

[8] Jankovic J . Treatment of dystonia. Lancet Neurol 2006;5(10):864–872. 16987733 10.1016/S1474-4422(06)70574-9

[9] Downs AM , Roman KM , Campbell SA , et al. The neurobiological basis for novel experimental therapeutics in dystonia. Neurobiol Dis 2019;130:104526. 31279827 10.1016/j.nbd.2019.104526 PMC6885011

[10] Pisani A , Bernardi G , Ding J , et al. Re‐emergence of striatal cholinergic interneurons in movement disorders. Trends Neurosci 2007;30(10):545–553. 17904652 10.1016/j.tins.2007.07.008

[11] Eskow Jaunarajs KL , Bonsi P , Chesselet MF , et al. Striatal cholinergic dysfunction as a unifying theme in the pathophysiology of dystonia. Prog Neurobiol 2015;127–128:91–107. 10.1016/j.pneurobio.2015.02.002 PMC4420693 25697043

[12] Scarduzio M , Zimmerman CN , Jaunarajs KL , et al. Strength of cholinergic tone dictates the polarity of dopamine D2 receptor modulation of striatal cholinergic interneuron excitability in DYT1 dystonia. Exp Neurol 2017;295:162–175. 28587876 10.1016/j.expneurol.2017.06.005 PMC5561742

[13] Ding JB , Guzman JN , Peterson JD , et al. Thalamic gating of corticostriatal signaling by cholinergic interneurons. Neuron 2010;67(2):294–307. 20670836 10.1016/j.neuron.2010.06.017 PMC4085694

[14] Sciamanna G , Tassone A , Mandolesi G , et al. Cholinergic dysfunction alters synaptic integration between thalamostriatal and corticostriatal inputs in DYT1 dystonia. J Neurosci 2012;32(35):11991–12004. 22933784 10.1523/JNEUROSCI.0041-12.2012 PMC3471539

[15] Clos MV , García‐Sanz A , Vivas NM , et al. D2 dopamine receptors and modulation of spontaneous acetylcholine (ACh) release from rat striatal synaptosomes. Br J Pharmacol 1997;122(2):286–290. 9313937 10.1038/sj.bjp.0701327 PMC1564915

[16] DeBoer P , Heeringa MJ , Abercrombie ED . Spontaneous release of acetylcholine in striatum is preferentially regulated by inhibitory dopamine D2 receptors. Eur J Pharmacol 1996;317(2–3):257–262. 8997608 10.1016/s0014-2999(96)00761-3

[17] Maurice N , Mercer J , Chan CS , et al. D2 dopamine receptor‐mediated modulation of voltage‐dependent Na+ channels reduces autonomous activity in striatal cholinergic interneurons. J Neurosci 2004;24(46):10289–10301. 15548642 10.1523/JNEUROSCI.2155-04.2004 PMC6730305

[18] Pisani A , Martella G , Tscherter A , et al. Altered responses to dopaminergic D2 receptor activation and N‐type calcium currents in striatal cholinergic interneurons in a mouse model of DYT1 dystonia. Neurobiol Dis 2006;24(2):318–325. 16934985 10.1016/j.nbd.2006.07.006

[19] Bonsi P , Ponterio G , Vanni V , et al. RGS9‐2 rescues dopamine D2 receptor levels and signaling in DYT1 dystonia mouse models. EMBO Mol Med 2019;11(1):e9283. 30552094 10.15252/emmm.201809283 PMC6328939

[20] Martella G , Tassone A , Sciamanna G , et al. Impairment of bidirectional synaptic plasticity in the striatum of a mouse model of DYT1 dystonia: role of endogenous acetylcholine. Brain 2009;132(Pt 9):2336–2349. 19641103 10.1093/brain/awp194 PMC2766181

[21] Martella G , Maltese M , Nisticò R , et al. Regional specificity of synaptic plasticity deficits in a knock‐in mouse model of DYT1 dystonia. Neurobiol Dis 2014;65:124–132. 24503369 10.1016/j.nbd.2014.01.016

[22] Grundmann K , Glöckle N , Martella G , et al. Generation of a novel rodent model for DYT1 dystonia. Neurobiol Dis 2012;47(1):61–74. 22472189 10.1016/j.nbd.2012.03.024

[23] Dang MT , Yokoi F , Cheetham CC , et al. An anticholinergic reverses motor control and corticostriatal LTD deficits in Dyt1 ΔGAG knock‐in mice. Behav Brain Res 2012;226(2):465–472. 21995941 10.1016/j.bbr.2011.10.002 PMC3225290

[24] Rittiner JE , Caffall ZF , Hernández‐Martinez R , et al. Functional genomic analyses of Mendelian and sporadic disease identify impaired eIF2α signaling as a generalizable mechanism for dystonia. Neuron 2016;92(6):1238–1251. 27939583 10.1016/j.neuron.2016.11.012 PMC5320521

[25] Maltese M , Martella G , Madeo G , et al. Anticholinergic drugs rescue synaptic plasticity in DYT1 dystonia: role of M1 muscarinic receptors. Mov Disord 2014;29(13):1655–1665. 25195914 10.1002/mds.26009 PMC4216601

[26] Goodchild RE , Kim CE , Dauer WT . Loss of the dystonia‐associated protein torsinA selectively disrupts the neuronal nuclear envelope. Neuron 2005;48(6):923–932. 16364897 10.1016/j.neuron.2005.11.010

[27] Imbriani P , Ponterio G , Tassone A , et al. Models of dystonia: an update. J Neurosci Methods 2020;339:108728. 32289333 10.1016/j.jneumeth.2020.108728

[28] Tassone A , Sciamanna G , Bonsi P , et al. Experimental models of dystonia. Int Rev Neurobiol 2011;98:551–572. 21907100 10.1016/B978-0-12-381328-2.00020-1

[29] Meringolo M , Tassone A , Imbriani P , et al. Dystonia: are animal models relevant in therapeutics? Rev Neurol 2018;174(9):608–614. 30153948 10.1016/j.neurol.2018.07.003

[30] Maltese M , Stanic J , Tassone A , et al. Early structural and functional plasticity alterations in a susceptibility period of DYT1 dystonia mouse striatum. Elife 2018;7:e33331. 29504938 10.7554/eLife.33331 PMC5849413

[31] Ponterio G , Tassone A , Sciamanna G , et al. Enhanced mu opioid receptor‐dependent opioidergic modulation of striatal cholinergic transmission in DYT1 dystonia. Mov Disord 2018;33(2):310–320. 29150865 10.1002/mds.27212

[32] Sciamanna G , Hollis R , Ball C , et al. Cholinergic dysregulation produced by selective inactivation of the dystonia‐associated protein torsinA. Neurobiol Dis 2012;47(3):416–427. 22579992 10.1016/j.nbd.2012.04.015 PMC3392411

[33] Vanni V , Puglisi F , Bonsi P , et al. Cerebellar synaptogenesis is compromised in mouse models of DYT1 dystonia. Exp Neurol 2015;271:457–467. 26183317 10.1016/j.expneurol.2015.07.005

[34] Livak KJ , Schmittgen TD . Analysis of relative gene expression data using real‐time quantitative PCR and the 2(‐Delta Delta C(T)) method. Methods 2001;25(4):402–408. 11846609 10.1006/meth.2001.1262

[35] Gangarossa G , Guzman M , Prado VF , et al. Role of the atypical vesicular glutamate transporter VGLUT3 in l‐DOPA‐induced dyskinesia. Neurobiol Dis 2016;87:69–79. 26711621 10.1016/j.nbd.2015.12.010

[36] Janickova H , Prado VF , Prado MAM , et al. Vesicular acetylcholine transporter (VAChT) over‐expression induces major modifications of striatal cholinergic interneuron morphology and function. J Neurochem 2017;142(6):857–875. 28628197 10.1111/jnc.14105

[37] Gras C , Amilhon B , Lepicard EM , et al. The vesicular glutamate transporter VGLUT3 synergizes striatal acetylcholine tone. Nat Neurosci 2008;11(3):292–300. 18278042 10.1038/nn2052

[38] El Mestikawy S , Wallén‐Mackenzie A , Fortin GM , et al. From glutamate co‐release to vesicular synergy: vesicular glutamate transporters. Nat Rev Neurosci 2011;12(4):204–216. 21415847 10.1038/nrn2969

[39] Sciamanna G , Tassone A , Martella G , et al. Developmental profile of the aberrant dopamine D2 receptor response in striatal cholinergic interneurons in DYT1 dystonia. PLoS One 2011;6(9):e24261. 21912682 10.1371/journal.pone.0024261 PMC3166312

[40] Hebb CO , Silver A . Gradient of choline acetylase activity. Nature 1961;189:123–125. 13712629 10.1038/189123a0

[41] Macintosh FC . The distribution of acetylcholine in the peripheral and the central nervous system. J Physiol (London) 1941;99(4):436–442. 16995263 10.1113/jphysiol.1941.sp003913 PMC1394098

[42] Bonsi P , Martella G , Cuomo D , et al. Loss of muscarinic autoreceptor function impairs long‐term depression but not long‐term potentiation in the striatum. J Neurosci 2008;28(24):6258–6263. 18550768 10.1523/JNEUROSCI.1678-08.2008 PMC3849426

[43] Goldberg JA , Ding JB , Surmeier DJ . Muscarinic modulation of striatal function and circuitry. Handb Exp Pharmacol 2012;208:223–241. 10.1007/978-3-642-23274-9_10 22222701

[44] Mamaligas AA , Ford CP . Spontaneous synaptic activation of muscarinic receptors by striatal cholinergic neuron firing. Neuron 2016;91(3):574–586. 27373830 10.1016/j.neuron.2016.06.021 PMC5234077

[45] Suzuki E , Momiyama T . M1 muscarinic acetylcholine receptor‐mediated inhibition of GABA release from striatal medium spiny neurons onto cholinergic interneurons. Eur J Neurosci 2021;53(3):796–813. 33270289 10.1111/ejn.15074

[46] Vogel C , Marcotte EM . Insights into the regulation of protein abundance from proteomic and transcriptomic analyses. Nat Rev Genet 2012;13(4):227–232. 22411467 10.1038/nrg3185 PMC3654667

[47] Guzman MS , De Jaeger X , Raulic S , et al. Elimination of the vesicular acetylcholine transporter in the striatum reveals regulation of behaviour by cholinergic‐glutamatergic co‐transmission. PLoS Biol 2011;9(11):e1001194. 22087075 10.1371/journal.pbio.1001194 PMC3210783

[48] Nagy PM , Aubert I . Overexpression of the vesicular acetylcholine transporter increased acetylcholine release in the hippocampus. Neuroscience 2012;218:1–11. 22641085 10.1016/j.neuroscience.2012.05.047

[49] Prado VF , Roy A , Kolisnyk B , et al. Regulation of cholinergic activity by the vesicular acetylcholine transporter. Biochem J 2013;450(2):265–274. 23410039 10.1042/BJ20121662

[50] Prado VF , Martins‐Silva C , de Castro BM , et al. Mice deficient for the vesicular acetylcholine transporter are myasthenic and have deficits in object and social recognition. Neuron 2006;51(5):601–612. 16950158 10.1016/j.neuron.2006.08.005

[51] Song C‐H , Bernhard D , Bolarinwa C , et al. Subtle microstructural changes of the striatum in a DYT1 knock‐in mouse model of dystonia. Neurobiol Dis 2013;54:362–371. 23336980 10.1016/j.nbd.2013.01.008 PMC3628999

[52] Bolam JP , Wainer BH , Smith AD . Characterization of cholinergic neurons in the rat neostriatum. A combination of choline acetyltransferase immunocytochemistry, Golgi‐impregnation and electron microscopy. Neuroscience 1984;12(3):711–718. 6382048 10.1016/0306-4522(84)90165-9

[53] Wilson CJ , Chang HT , Kitai ST . Firing patterns and synaptic potentials of identified giant aspiny interneurons in the rat neostriatum. J Neurosci 1990;10(2):508–519. 2303856 10.1523/JNEUROSCI.10-02-00508.1990 PMC6570144

[54] Wilson CJ . The mechanism of intrinsic amplification of hyperpolarizations and spontaneous bursting in striatal cholinergic interneurons. Neuron 2005;45(4):575–585. 15721243 10.1016/j.neuron.2004.12.053

[55] Liu Y , Xing H , Sheng W , et al. Alteration of the cholinergic system and motor deficits in cholinergic neuron‐specific Dyt1 knockout mice. Neurobiol Dis 2021;2021:105342. 10.1016/j.nbd.2021.105342 PMC8113083 33757902

[56] Eskow Jaunarajs KL , Scarduzio M , Ehrlich ME , et al. Diverse mechanisms lead to common dysfunction of striatal cholinergic interneurons in distinct genetic mouse models of dystonia. J Neurosci 2019;39(36):7195–7205. 31320448 10.1523/JNEUROSCI.0407-19.2019 PMC6733543

[57] Dong GZ , Kameyama K , Rinken A , Haga T . Ligand binding properties of muscarinic acetylcholine receptor subtypes (m1‐m5) expressed in baculovirus‐infected insect cells. J Pharmacol Exp Ther 1995;274(1):378–384. 7616422

[58] Augood SJ , Martin DM , Ozelius LJ , et al. Distribution of the mRNAs encoding torsinA and torsinB in the normal adult human brain. Ann Neurol 1999;46(5):761–769. 10553994

[59] Pappas SS , Darr K , Holley SM , et al. Forebrain deletion of the dystonia protein torsinA causes dystonic‐like movements and loss of striatal cholinergic neurons. Elife 2015;4:e08352. 26052670 10.7554/eLife.08352 PMC4473728

[60] Yokoi F , Oleas J , Xing H , et al. Decreased number of striatal cholinergic interneurons and motor deficits in dopamine receptor 2‐expressing‐cell‐specific Dyt1 conditional knockout mice. Neurobiol Dis 2020;134:104638. 31618684 10.1016/j.nbd.2019.104638 PMC7323754

[61] McNaught KSP , Kapustin A , Jackson T , et al. Brainstem pathology in DYT1 primary torsion dystonia. Ann Neurol 2004;56(4):540–547. 15455404 10.1002/ana.20225

[62] Pratt D , Mente K , Rahimpour S , et al. Diminishing evidence for torsinA‐positive neuronal inclusions in DYT1 dystonia. Acta Neuropathol Commun 2016;4(1):85. 27531128 10.1186/s40478-016-0362-z PMC4988029

[63] Sharma N . Neuropathology of dystonia. Tremor Other Hyperkinet Mov (NY) 2019;9:569. 10.7916/d8-j6sx-b156 PMC6420908 30886764

[64] Giboureau N , Som IM , Boucher‐Arnold A , et al. PET radioligands for the vesicular acetylcholine transporter (VAChT). Curr Top Med Chem 2010;10(15):1569–1583. 20583990 10.2174/156802610793176846

[65] Bohnen NI , Kanel P , Zhou Z , et al. Cholinergic system changes of falls and freezing of gait in Parkinson's disease. Ann Neurol 2019;85(4):538–549. 30720884 10.1002/ana.25430 PMC6450746

[66] Mazere J , Dilharreguy B , Catheline G , et al. Striatal and cerebellar vesicular acetylcholine transporter expression is disrupted in human DYT1 dystonia. Brain 2021;144(3):909–923. 33638639 10.1093/brain/awaa465

[67] Albin RL , Cross D , Cornblath WT , et al. Diminished striatal [123I]iodobenzovesamicol binding in idiopathic cervical dystonia. Ann Neurol 2003;53(4):528–532. 12666122 10.1002/ana.10527

